# NGS in the clinical microbiology settings

**DOI:** 10.3389/fcimb.2022.955481

**Published:** 2022-10-19

**Authors:** Milena Pitashny, Balqees Kadry, Raya Shalaginov, Liat Gazit, Yaniv Zohar, Moran Szwarcwort, Yoav Stabholz, Mical Paul

**Affiliations:** ^1^ Clinical and Research Microbiome Center, Research Division, Rambam Health Care Campus, Haifa, Israel; ^2^ Clinical Microbiology Laboratories, Laboratories Division, Rambam Health Care Campus, Haifa, Israel; ^3^ Pathology Institute, Rambam Health Care Campus, Haifa, Israel; ^4^ Infectious Diseases Unit, Rambam Health Care Campus, Haifa, Israel

**Keywords:** NGS, 16s, clinical microbiology, next generation sequencing, polymicrobial infections, polymicrobial

## Abstract

We hypothesized that targeted NGS sequencing might have an advantage over Sanger sequencing, especially in polymicrobial infections. The study included 55 specimens from 51 patients. We compared targeted NGS to Sanger sequencing in clinical samples submitted for Sanger sequencing. The overall concordance rate was 58% (32/55) for NGS vs. Sanger. NGS identified 9 polymicrobial and 2 monomicrobial infections among 19 Sanger-negative samples and 8 polymicrobial infections in 11 samples where a 16S gene was identified by gel electrophoresis, but could not be mapped to an identified pathogen by Sanger. We estimated that NGS could have contributed to patient management in 6/18 evaluated patients and thus has an advantage over Sanger sequencing in certain polymicrobial infections.

## Introduction

Most clinical infections are not microbiologically confirmed. The problem is especially pertinent in deep seated invasive infections, where microbiological diagnosis is critical and the specimen is precious, such as osteomyelitis, deep organ abscesses, brain empyema or others. For these cases, there has been a growing interest and implementation of broad-range polymerase chain reaction (PCR) based Sanger sequencing of the 16S ribosomal RNA (rRNA) bacterial gene (panbacterial PCR), directly from clinical specimens (Rampini et al., 2011). Sanger sequencing significantly improved the diagnostic yield in clinical culture isolates as well as mono-microbial infections (Shachor-Meyouhas et al., 2013; Khoury et al., 2019). However, when more than one species are present in the specimen, Sanger sequencing template reads are superimposed and are generally uninterpretable (Salipante et al., 2013). Results from such specimens are reported as negative or as a mixture of bacteria, without further identification.

As in other fields of medicine, next generation sequencing (NGS) technologies have expanded diagnostic capabilities in clinical microbiology laboratories. Recent studies have highlighted the ability of 16S rRNA NGS to accurately reach speciation and quantify bacterial abundances in complex polymicrobial infections (Salipante et al., 2013; Abayasekara et al., 2017; Culbreath et al., 2019).

We hypothesized that for difficult-to-diagnose infections, especially when polymicrobial, targeted NGS of the 16S rRNA gene has better diagnostic performance than panbacterial Sanger sequencing.

## Materials and methods

We used residual nucleic acids from clinical specimens that were submitted to our reference molecular laboratory at RHCC for panbacterial, panfungal or mycobacterial PCR (Sanger sequencing). Samples were collected between 2020-2021 at Rambam Health Care Campus (RHCC) or other hospitals. DNA stored at -20oC was retrieved and tested by NGS retrospectively. Clinical information was available only for samples sent from within RHCC.

Broad-range 16S rRNA gene Sanger sequencing was performed using in-house protocols at the molecular bacteriology laboratory (Shachor-Meyouhas et al., 2013).

NGS library preparation and analyses were performed blinded to clinical information, culture or Sanger sequencing results. DNA extraction of bacterial DNA from each specimen was carried out using the QIAAmp^®^ DNA Mini kit (QiagenGroup) according to manufacturer’s instructions. Each batch of specimens were extracted with negative controls (extraction control). PCR amplification of the hypervariable region V4 of the 16s rRNA gene was conducted using PCRBIO HS Taq Mix Red according to the Earth Microbiome Project primer pairs (Apprill et al., 2015; Parada et al., 2016), and in selected cases, the addition of V1-2 segments (F27-R338) of the same gene to better identify certain bacteria and improve speciation (such as for Staphylococcus and Enterobacterales) (Walker et al., 2015). PCR products were run on a 1.5% agarose gel. Final library products were purified using Qiaquick PCR Purification Kit (Qiagen Groups) according to manufacturer’s instructions and quantified using Qubit™ dsDNA HS and BR Assay Kits (Invitrogen).

We sequenced the amplified V1-2 or V4 regions of 16s rRNA gene with Ion S5™ System (Thermo Fisher Scientific). Data were analyzed using the Ion Reporter bioinformatics Software pipeline (Thermo Fisher Scientific), using a threshold of 1000 mapped reads for designating significant pathogens. BAM files uploaded to the Ion Reporter were mapped to the Silva 138 SSU database. Typical contaminants found in negative controls, such as Acinetobacter lwofii, Acinetobacter schendleri, or Xanthomonadaceae which are water tolerant bacteria, were subtracted from overall reads obtained on the clinical samples.

Concordance between Sanger sequencing and NGS results was evaluated. For the RHCC samples, we estimated the potential clinical added value of NGS, had it been available in real time. Two physicians (infectious diseases and clinical microbiologist) evaluated each case and assigned the potential contribution of NGS to patient management independently (diagnosis and treatment). Disagreements were resolved by consensus.

The study was approved by the local ethics committee with a waiver of informed consent. NGS results were not conveyed to clinicians.

## Results

**Figure 1:**
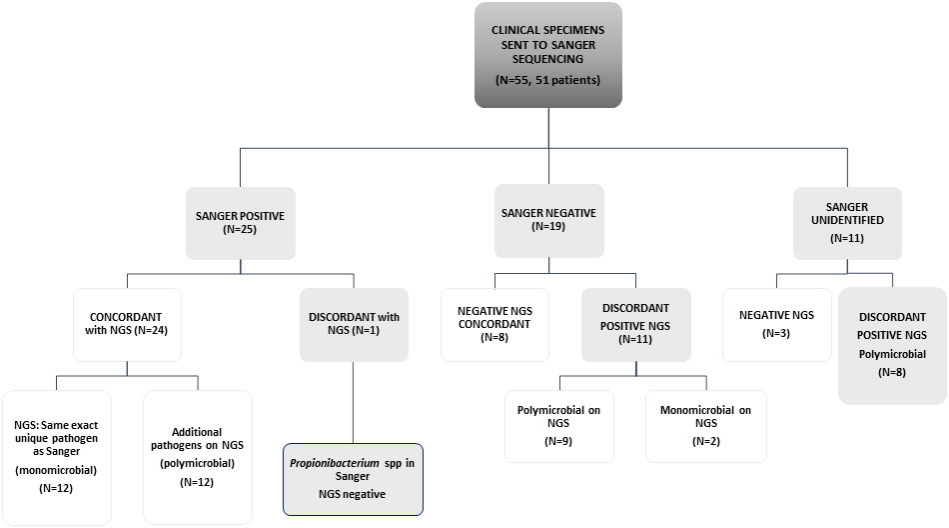
Concordance between Sanger sequencing and NGS in a scheme. Sanger negative are those samples that did not give any signal on PCR gels. "Sanger unidentified" are those samples that presented a band in agarose gels, but on Sanger sequencing it was not possible to define a unique organism against public databases.

The study included 55 specimens from 51 patients; 22 specimens from RHCC and all others from other hospitals. Of the 55 specimens evaluated, 25 specimens were Sanger positive with one organism reported. Of those, 24 were concordant by NGS (24/25) that identified the exact Sanger pathogen alone (N=12) or with additional pathogen/s (N=12). The only discordant result was a *Propionibacterium* spp identified by Sanger, that was missed by NGS. Sanger was negative in 19 samples, of which 8 were negative by NGS, 9 polymicrobial and 2 monomicrobial by NGS. In 11 samples, broad-range 16S gene was identified by gel electrophoresis, but could not be mapped to an identified pathogen within available databases when sequenced by Sanger technology (possible polymicrobial infection); among these, 3/11 were negative on NGS and all others were positive with polymicrobial identification (**Figure 1** and **Table 1**).

**Table 1:** Comparison between Sanger and NGS results.

Sample ID	Material	Sanger results	NGS results	Total number of mapped reads
**Concordant, positive** N=24				
NGS-1	Tissue/skin/soft tissue	*Pseudomonas aeruginosa*	*Pseudomonas* spp 28% (*Pseudomonas aeruginosa* 2%)	7946
NGS-14	Pus	*Pseudomonas aeruginosa*	*Pseudomonas* 74% *(P.aeruginosa* 39%*)*	18412
NGS-15	Pus	*Fusobacterium necrophorum*	*Fusobacterium necrophorum* 34%	16616
NGS-2	Wound	*Mycobacterium marinum*	*Mycobacterium spp* 20% V1-2: *Mycobacterium marinum*	4208
NGS-20	Tissue	*Staphylococcus aureus*	*Staphylococcus* spp 71%(*S. epidermidis*11%, *S. aureus* 3%) V1-2: *S.aureus* 37%	9765
NGS-3	Tissue	*Streptococcus pyogenes*	*Streptococcus pyogenes*27%	4929
NGS-33	Blood	*Sneathia sanguinegens*	*Sneathia sanguinegens 60%*	201717
NGS-39	Tissue	*Staphylococcus aureus*	*Staphylococcus* spp 72% (*S. epidermidis* 9% *, S.aureus* (1.5%)) V1-2: *S.aureus* 63% (low counts 446)	2609
NGS-40	lymph node	*Streptococcus pyogenes*	*Streptococcus pyogenes 35%*	1440
NGS-46	Pleural Fluid	*Enterococcus spp*	*Enterococcus* spp 29% (*E. moraviensis* 1%)	1569
NGS-7	Tissue	*Streptococcus dysgalactiae spp equisimilis*	*Streptococcus dysgalactiae 30%*	13866
NGS-9	Tissue	*Staphylococcus aureus*	*Staphylococcus* spp 41% (*S. epidermidis* 4% ) V1-2: *Staphylococcus aureus 33%*	2107
NGS-32	Swab	*Streptococcus sanguis*	Polymicrobial (*Rothia mucilaginosa* 10%, *Velionella dispar* 12%, *Prevotella* 11% (P. *histicola* 5%, *P.salivae* 3%, others <1%) *Streptococcus* 35%, (*S. australis* 11%, S. *infantis* 3%, S *thermophilus*1%), *Actinobacillus parahemolyticus* 2%)	2038
NGS-36	BAL	Streptococcus mitis	Polymicrobial (*Rothia mucilaginosa* 28%, *Streptococcus* 46% (*S. australis* 6%, S. *infantis* 8%), *Velionella* <1%) V1-2: (*Streptococcus pneumonia 2% and Pseudomonas aeruginosa* 3% in low counts 1059)	4870
NGS-53	Swab	*Haemophilus influenzae*	*Haemophilus* 35% (*H. influenzae* 20%), *Peptostreptococcus anaerobius* 2%	1737
NGS-11	Fluid	*Porphyromonas spp*	Polymicrobial (*Porphyromonas endodontalis* 25%), *Bacteroides fragilis* 5%)	10198
NGS-12	Pus	*Enterobacteriaceae*	Polymicrobial (*Enterobacter 3%, Klebsiella 2% , Enterococcus 3%* )	20144
NGS-25	Bronchial wash	*Pseudomonas spp*	Polymicrobial (*Prevotella* 15% (*P. histicola*11%, *P.melaninogenica*3%, *P.veroralis* <1%), *Velionella dispar* 7%, *Pseudomonas* spp 16%, *Streptococcus* spp 23 (*S.infantis 1%anginosus, australis, thermophilus* <1%) (V4) V1-2: *Streptococcus 7% S. salivarius, S.mitis*	54561
NGS-44	Abscess	*Enterobacteriaceae*	*Salmonella 5%, Escherichia 2%*	699
NGS-49	Tissue	*Proteus spp*	Polymicrobial (*Peptoniphilus 23%, Finegoldia magna 1%)* V1-2: *Proteus mirabilis 40%, Enterococcus faecalis 4%, Morganella morganii 17%*	889
NGS-50	Tissue	*Finegoldia spp*	Polymicrobial (*Peptoniphilus* 9%, *Finegoldia magna* 23%, *Prevotella timonensis* 10%, *Anaeroccocus murdochi*i 5%)	1614
NGS-51	Swab	*Capnocytophaga spp*	Polymicrobial (*Granulicatella adiacens 1%, Capnocytophaga leadbetteri 8% , Fusobacterium periodonticum<1%, Rothia mucilaginosa 4%, Neisseria cinerea15%, Haemophilus parainfluenzae 8%, Prevotella nanceiensis 3%, Streptococcus australis , Streptococcus infantis , Veillonella*)	7495
NGS-6	Pus	*Morganella spp*	Polymicrobial (*Pseudomonas spp 39% (Pseudomonas aeruginosa 16%)* V1-2: *Morganella morgani 25%*	4772
NGS-8	Pus	*Prevotella spp*	Polymicrobial (*Prevotella melaninogenica 19%, Finegoldia magna 10%,Vellionella spp 10%, Gemella spp 4%*)	18344
**Concordant, negative (N=8)**				
NGS-18	Tissue	Negative	Negative	551
NGS-21	Synovial Fluid	Negative	Negative	46968
NGS-22	Synovial Fluid	Negative	Negative	29735
NGS-24	Tissue biopsy, abscess	Negative	Negative	0
NGS-29	CSF, surgical site	Negative	Negative	428
NGS-35	CSF	Negative	Negative	494
NGS-43	Fluid	Negative	Negative	900
NGS-52	CSF	Negative	Negative	1211
**Discordant, Sanger-negative,** N=11				
NGS-13	Tissue	Negative	Polymicrobial (*Streptococcus 20% S. infantis 1%, S. australis 1%, Granulicatella elegans 2%, Gemella spp 4%, Haemophilus parainfluenza 3%, Neisseria 1%, Rothia mucilaginosa 2%, Prevotella melaninogenica 6%*) *V1-2*: *Helicobacter pylori 19%*	7668
NGS-16	Synovial fluid	Negative	Polymicrobial (*Porphyromonas uenonis 3%, Prevotella oris 6%, Prevoltella oralis 2%, Parvimonas micra 5%, Eubacterium infirmum 3%, Fusobacterium 5%*)	9467
NGS-17	Pleural fluid	Negative	Polymicrobial (*Prevotella oris 1%, Parvimonas micra 1%, Fusobacterium 3%*)	2253
NGS-19	Tissue	Negative	*Polymicrobial (Porphyromonas uenonis 1%, Parvimonas micra 2%, Fusobacterium 2%, Prevotella 2% oris and oralis)*	4612
NGS-23	Abscess	Negative	*Staphylococcus 20% (S.aureus <1%, S. epidermidis 4%)* *V1-2:* *Staphylococcus aureus 20%*	32376
NGS-26	CSF	Negative	Polymicrobial *(Staphylococcus 21% ( S. epidermidis 2%), Micrococcus 2%, Paenibacillus 5%, Legionella pneumophila 1.6%)* *V1-2:* *Legionella pneumophila 22%*	3753
NGS-27	Tissue-brain	Negative	*Staphylococcus 22% ( S. epidermidis 2%)*	3482
NGS-28	Tissue	Negative	Polymicrobial (*Corynebacterium spp 10%, Dermatobacter hominis 2%, Anaerococcus murdochii 2%, Peptoniphilus 9%, Clostridium ramosum <1%*)	8514
NGS-30	Tissue biopsy	Negative	Polymicrobial (*Corynebacterium kroppenstedtii <1%, Enterobacteriaceae 5% (Enterobacter cloacae <1%)*)	5123
NGS-4	Wound swab	Negative	Polymicrobial (*Prevotella oralis* 1%, *Prevotella oris* 3%. *Parvimonas micra* 3%, *Fusobacterium* 9% (*F. nucleatum* <1%), *Pseudomonas* 2% (*P.auruginosa* <1%, *P.hibiscicola* <1%)	8376
NGS-55	BAL	Negative	*Streptococcus* 40% (*S. infantis* 5%) V1-2 *(very low counts 191):* *Haemophilus parainfluenza*	1175
**Discordant, Sanger-positive, N=1**				
NGS-42	Surgical wound/abscess	*Propionibacterium spp*	Negative	504
**Sanger positive but unidentified (N=11)**				
NGS-31	Pus	Positive, unidentified	Negative	817
NGS-38	BAL	Positive, unidentified	Negative	458
NGS-45	BAL	Positive, unidentified	Negative	551
NGS-10	Tissue	Positive, unidentified	Polymicrobial (Finegoldia magna 18%, Enterococcus 11%, Bacteroides 21% ( B. ovatus 7, uniformis 5%), Parabacteroides 8% ( P. diastasonis4%), Corynebacterium 1% (C. jeikeium & tuberculostearicum <1%)	11542
NGS-34	Synovial fluid/ bone/ tissue	Positive, unidentified	Polymicrobial (V4 negative-only contaminants. *V1-2 (3890):* *E.coli 1%, Brebundimonas nasdae 2%, Microbacterium chocolatum 2%, Acinetobacter hemolyticus 1%*)	12452
NGS-37	Urethral secretion	Positive, unidentified	Polymicrobial (*Ureaplasma 27%, Corynebacterium tuberculostearicum 6%, Haemophilus Aegyptus 3%, Haemophilus influenza 3% , Staphylococcus 3%, Streptococcus 18% (S. infantis 3%)*) V1-2: *Ureaplasma urealyticum* 30%	7502
NGS-41	BAL	Positive, unidentified	*Pseudomonas* 42% (*P. auruginosa* 14%), *Streptococcus* 44% (*S. infantis* 4%)	3731
NGS-47	BAL	Positive, unidentified	*Prevotella 33% (P.nanceiensis 16%)*	1296
NGS-48	Tissue	Positive, unidentified	Polymicrobial (*Corynebacterium 7%, Campylobacter ureolyticus 7%, Anaerococcus vaginalis 6%, Finegoldia magna 10%, Peptoniphilus2%, Peptoniphilus 2%, Prevotella corporis7%, Streptococcus infantis <1%* , *Haemophilus 5% (H. aegyptius 3%, H. influenza 2%*) V1-2 (2121): *Moraxella catarrhalis 6%*	8234
NGS-5	Tissue	Positive, unidentified	Polymicrobial (*Pseudomonas spp 31% (P. aeruginosa 13%) Enterobacteriaceae 10% (Salmonella enterica 2.5%)*)	2756
NGS-54	BAL	Positive, unidentified	*Streptococcus 38% (S. infanti 5%)*	1318

Each specimen sent to the Clinical Microbiology Laboratory at Rambam for Sanger sequencing, was later sequenced with Ion torrent S5 for the amplification of the V4 hypervariable region of 16s ribosomal gene. Unless noted in the NGS results column, all sequences were found with the primer set V4. Whenever the addition of the variable region V1-2 gave further characterization, it was noted in this table. In the last column , the number of mapped reads with V4. In the NGS results column, the percentage of those reads attributed to each organism.

**Figure 2:**
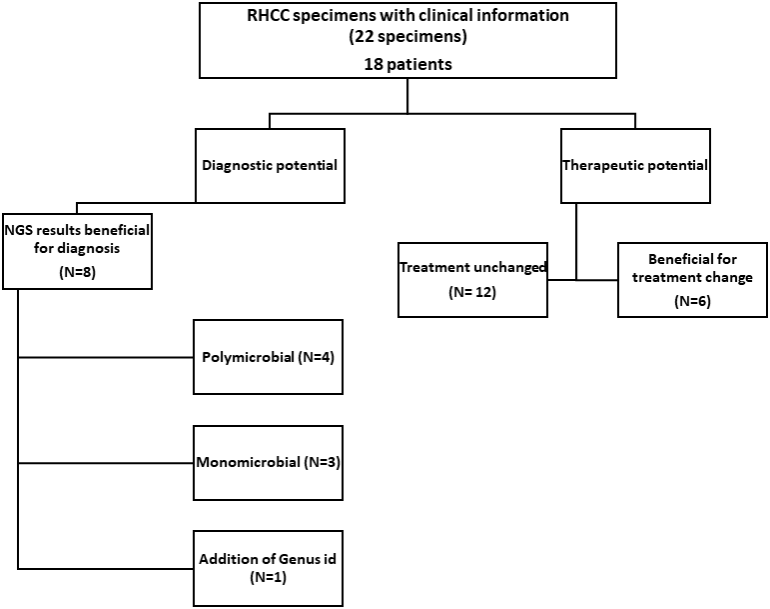
Effect of NGS findings on patient management. In 8/22 samples the result obtained with NGS contributed to diagnosis (also congruent when there were repeated samples from the same patient). In 6/18 patients, treatment could have been changed to a more appropriate one had NGS results been available at the time of diagnosis.

Among the 22 RHCC specimens for which clinical information was available (18 patients), eight could have benefited from NGS for diagnosis (**Figure 2** and **Table 2**): 4 polymicrobial (NGS-26, 30, 32, 34), 3 monomicrobial findings (NGS-23, 27, 54) and 1 genus identification of *E.coli/Salmonella* spp. in specimen NGS-44. We estimated that 6/18 patients would have benefited from antibiotic adjustment following NGS results and they all belong to the discordant group (**Figure 2** and **Table 2**), while the other 12 patients would have been treated properly without any change in empiric treatment.

**Table 2:** Potential added value of NGS over Sanger for patient management.

Sample ID	Diagnosis	Sanger	NGS results	Would have affected diagnosis?	Antimicrobial treatment	Would have changed treatment?
**Discordant **						
NGS-30	Chronic internal fixation-associated infection	Negative	Polymicrobial (*Corynebacterium kroppenstedtii, Enterobacteriaceae, Enterobacter cloacae*)	Yes	Cefazolin+ Ciprofloxacin	Possibly
NGS-34	Postpartum sacroiliac joint arthritis	Positive, unidentified	Polymicrobial (*E.coli, Brebundimonas nasdae, Microbacterium chocolatum, Acinetobacter hemolyticus*)	Yes (See NGS-33)	Piperacillin-Tazobactam, subsq: Metronidazole and then Amoxicillin	Yes
NGS-32	Necrotizing cervical lymph node (HIV positive)	*Streptococcus sanguis*	Polymicrobial (*Rothia mucilaginosa, Velionella dispar, Prevotella histicola, Prevotella salivae, Streptococcus australis, Streptococcus infantis, Actinobacillus parahemolyticus, Haemophilus parahemolyticus*)	Yes (See NGS-31)	Unknown (No EMR access)	Possibly
NGS-26	Nosocomial meningitis	Negative	Polymicrobial (*Legionella pneumophila, Staphylococcus epidermidis*)	Yes	Meropenem+ Vancomycin	Possibly
NGS-27	Nosocomial meningitis	Negative	*Staphylococcus epidermidis*	Yes (See NGS-26)	Meropenem+ Vancomycin	Possibly
NGS-23	Chronic osteomyelitis with Leg abscess	Negative	*Staphylococcus aureus*	Yes	Cefazolin	No
NGS-54	Cavitary pneumonia (congenital neutropenia)	Positive, unidentified	*Streptococcus salivarius*	Yes (See NGS-25)	Meropenem	Possibly
NGS-44	Arm fluctuant lesion (AML)	*Enterobacteriaceae*	*Salmonella, Escherichia*	Yes	Amoxicillin-clavulanate	Possibly
NGS-25	Cavitary pneumonia (congenital neutropenia)	*Pseudomonas spp*	Polymicrobial (*Streptococcus salivarius, mitis, anginosus, Prevotella melaninogenica, Velionella dispar , Pseudomonas aeruginosa*)	No. Positive culture	Piperacillin-Tazobactam Subsq. Levofloxacin	No
NGS-41	Hilar lymphadenopathy (AML)	Positive, unidentified	*Pseudomonas aeruginosa*	No. Positive culture	Levofloxacin	No
NGS-4	Jaw lesion (AML)	Negative	Polymicrobial (*Prevotella oralis, Prevotella oris, Parvimonas micra, Fusobacterium nucleatum, Pseudomonas aeruginosa, P.hibiscicola*)	No. Mucormycosis	Amphotericin-B + Posaconazole	No
**Concordant **						
NGS-1	Leg ulcer	*Pseudomonas aeruginosa*	*Pseudomonas aeruginosa*	No		No
NGS-2	Left hand abscess	*Mycobacterium marinum*	*Mycobacterium marinum*	No		No
NGS-3	Leg cellulitis	*Streptococcus pyogenes*	*Streptococcus pyogenes*	No		No
NGS-33	Postpartum sacroiliac joint arthritis	*Sneathia sanguinegens*	*Sneathia sanguinegens*	No		No
NGS-21	Suspected septic arthritis of hip	Negative	Negative	No		No
NGS-24	Synovitis	Negative	Negative	No		No
NGS-29	Nosocomial Meningitis	Negative	Negative	No		No
NGS-31	Necrotizing cervical lymph node (HIV positive)	Positive, unidentified	Negative	No		No
NGS-35	Suspected Brain abscess and meningitis	Negative	Negative	No		No
NGS-42	Deep neurosurgical site infection	*Propionibacterium spp*	Negative	No		No

Available clinical data was collected for samples sent from within Rambam. Retrospectively, a Clinical Microbiologist and an Infectious diseases specialist independently evaluated the diagnostic potential of NGS over Sanger and the potential for therapeutic modifications. Incongruences in the conclusions were solved by the two specialists by consensus.

In this study we describe the comparison between broad range Sanger panbacterial sequencing and targeted deep sequencing (NGS) on clinical samples submitted for panbacterial PCR. Overall, the concordance rate was 58% (32/55) for NGS vs. Sanger. Concordance was more frequent in Sanger-positive samples 24/25 (96%) than for Sanger-negative 8/19 (42%). Among five discordant Sanger-negative results with clinical information (**Table 2**), the positive NGS result would have been considered clinically-significant and might have improved diagnosis and/or management. In addition, NGS was able to identify possible pathogens in 8/11 Sanger-positive but pathogen-unidentified specimens. These results in a diagnostic advantage to targeted NGS. Moreover, polymicrobial communities identified by NGS may point to particular infection processes that may contribute to patients’ evaluation and optimal management. This is consistent with previous studies that validated the use of NGS for pathogen detection (Culbreath et al., 2019) and compared NGS to culture-based diagnosis (Abayasekara et al., 2017).

## Discussion

The targeted NGS in-house assay was performed on the Ion torrent S5 instrument, used for microbiome purposes, with a predefined threshold of > 1000 mapped reads. The presence of certain pathogens, however, should always be considered as a potential cause of infection, even if the number of reads is below predefined cutoffs, in polymicrobial or monomicrobial results. Such is the case in specimens NGS-44 (699 mapped reads) where *Salmonella* identified by NGS was considered clinically -significant, and NGS-49 (889 reads) where *Proteus mirabilis* magna among other intestinal pathogens might have been clinically-significant. Conversely, the presence of common commensals should be interpreted carefully. The clinician and the clinical microbiologist must work together to attribute clinical significance to the NGS results.

NGS technology allows for the parallel coverage of all taxa present in a clinical specimen, resulting in the identification of complex microbial communities. The polymicrobial findings in our study likely represented polymicrobial infections. However, alternative explanations should be considered, such as the possibility of commensal microbiota present in non-sterile or sterile body sites, a non-sterile specimen collection technique, or contamination during laboratory workflows. To overcome the latter, species found in negative controls were considered contaminants in our study and their sequences were subtracted from results. During specimen collection and transportation, polymicrobial communities may change in composition. Sanger identifies the best amplified organism which is not necessarily representative of the dominant or pathogenic one

One central limitation of this study is that only bacterial organisms were targeted (V4 and V1-2 regions of 16S rRNA gene). In addition, samples were selected randomly for this analysis, but not consecutively. The study was non-interventional – NGS results were not used in clinical practice, thus its true effect on patient management remain unknown. One advantage of this study is that NGS was performed blinded to other microbiological and clinical information.

In conclusion, in this validation study we demonstrated superior pathogen identification with targeted 16s NGS compared to Sanger sequencing in clinical samples. We propose to consider NGS upfront in cases where polymicrobial infections are suspected. Further developments of NGS should include the addition of other important targets such as viral targets or Internal Transcribed Space (ITS) for fungi, as well as antimicrobial resistance genes. To better characterize the accuracy of results, comparison with shotgun metagenomics is necessary.

## Data availability statement

The original contributions presented in the study are publicly available. This data can be found here: [DOI: 10.5281/zenodo.7119981].

## Ethics statement

The studies involving human participants were reviewed and approved by Helsinki committee at Rambam HCC, Israel. Written informed consent for participation was not required for this study in accordance with the national legislation and the institutional requirements.

## Author contributions

Authors’ contributions: All authors contributed to the study conception and design. Material preparation, data collection and analysis were performed by MPi, BK, and YS. The first draft of the manuscript was written by MPi and all authors commented on previous versions of the manuscript. All authors read and approved the final manuscript.

## Conflict of interest

The authors declare that the research was conducted in the absence of any commercial or financial relationships that could be construed as a potential conflict of interest.

## Publisher’s note

All claims expressed in this article are solely those of the authors and do not necessarily represent those of their affiliated organizations, or those of the publisher, the editors and the reviewers. Any product that may be evaluated in this article, or claim that may be made by its manufacturer, is not guaranteed or endorsed by the publisher.

## References

[B1] Abayasekara L. M. Perera J. Chandrasekharan V. Gnanam V. S. Udunuwara N. A. Liyanage D. S.. (2017). Detection of bacterial pathogens from clinical specimens using conventional microbial culture and 16S metagenomics: a comparative study. BMC Infect. Dis. 17 (1), 631. 28927397 10.1186/s12879-017-2727-8 PMC5606128

[B2] Apprill A. Mcnally S. Parsons R. Weber L. (2015). Minor revision to V4 region SSU rRNA 806R gene primer greatly increases detection of SAR11 bacterioplankton. Aquat Microb. Ecol. 75 (2), 129–137.

[B3] Culbreath K. Melanson S. Gale J. Baker J. Li F. Saebo O.. (2019). Validation and retrospective clinical evaluation of a quantitative 16S rRNA gene metagenomic sequencing assay for bacterial pathogen detection in body fluids. J. Mol. Diagn. 21 (5), 913–923. 31229651 10.1016/j.jmoldx.2019.05.002

[B4] Khoury N. Amit S. Geffen Y. Adler A. (2019). Clinical utility of pan-microbial PCR assays in the routine diagnosis of infectious diseases. Diagn. Microbiol. Infect. Dis. 93 (3), 232–237. 30509499 10.1016/j.diagmicrobio.2018.09.016

[B5] Parada A. E. Needham D. M. Fuhrman J. A. (2016). Every base matters: assessing small subunit rRNA primers for marine microbiomes with mock communities, time series and global field samples. Environ. Microbiol. 18 (5), 1403–1414. doi: 10.1111/1462-2920.13023 26271760

[B6] Rampini S. K. Bloemberg G. V. Keller P. M. Büchler A. C. Dollenmaier G. Speck R. F.. (2011). Broad-range 16S rRNA gene polymerase chain reaction for diagnosis of culture-negative bacterial infections. Clin. Infect. Dis. 53 (12), 1245–1251. 21976460 10.1093/cid/cir692

[B7] Salipante S. J. Sengupta D. J. Rosenthal C. Costa G. Spangler J. Sims E. H.. (2013). Rapid 16S rRNA next-generation sequencing of polymicrobial clinical samples for diagnosis of complex bacterial infections. PloS One 8 (5), e65226. 23734239 10.1371/journal.pone.0065226 PMC3666980

[B8] Shachor-Meyouhas Y. Sprecher H. Moscoviz D. Zaidman I. Haimi M. Kassis I. (2013). Molecular-based diagnosis of bacteremia in the setting of fever with or without neutropenia in pediatric hematology-oncology patients. J. Pediatr. Hematol. Oncol. 35 (7), 500–503. 24064965 10.1097/MPH.0b013e31829eec78

[B9] Walker A. W. Martin J. C. Scott P. Parkhill J. Flint H. J. Scott K. P. (2015). 16S rRNA gene-based profiling of the human infant gut microbiota is strongly influenced by sample processing and PCR primer choice. Microbiome 3 (1), 1–11. doi: 10.1186/s40168-015-0087-4 26120470 PMC4482049

